# HPB News

**DOI:** 10.1155/1990/42670

**Published:** 1990


					HPB Surgery, 1990, Vol. 2, p. 81
Reprints available directly from the publisher
Photocopying permitted by license only
1990 Harwood Academic Publishers GmbH
Printed in the United Kingdom
HPB NEWS
IHBPA announces its 12th meeting to be organized in Hongkong August 22-24,
1990. As previously, the meeting will offer invited lecturers, symposia, consensus,
panel discussions, free papers, poster sessions and video presentations. The
following topics are given priority: liver and bile ducts anatomy/interoperative
ultrasound, the large common duct stone, hepatocellular carcinoma: risk factors,
diagnosis and therapy, hepatolithiasis, intrahepatic bile duct disorders, liver trans-
plantation harvesting techniques, non-surgical complications, portal
hypertension/oesophageal varic6s, acute necrotizing pancreatitis, occasions of pan-
creatic surgery, assessment of exocrine pancreatic function.
The deadline for submission of abstracts is February 1, 1990, for registration
(lower registration fee) is April 1, 1990. The registration fees are: non-members
USD 400/450, IHPBA members 350/400, trainees 150/200, accompanying persons
100-150. The programme committee consists of J. Toouli, Australia chairman;
D. Nagorney, USA; F. Nakayama, Japan; E. Seifert, West Germany and J. Wong,
Hongkong.
Information can be obtained from the secretariat of the conference. Write or
call: IHBPA Hongkong, Department of Surgery, University of Hongkong, Queen
Mary Hospital, Hongkong, telephone: int + 852 (5) 819 2235 or fax int + 852
(5) 855 1897.
81

				

